# Fluorescence of Cladophialophora Bantiana After Administration of 5-Aminolevulinic Acid Hydrochloride: Case Report

**DOI:** 10.1227/neuprac.0000000000000164

**Published:** 2025-09-24

**Authors:** Macy Mitchell, Bobbi Thursam, Nicholas Figueroa, Bryan Figueroa

**Affiliations:** * Michigan State University College of Human Medicine, Grand Rapids, Michigan, USA;; ‡ Department of Neurology, Trinity Health West Michigan, Grand Rapids, Michigan, USA;; § Hope College, Holland, Michigan, USA;; ‖ Great Lakes Neurosurgical Associates PC, Grand Rapids, Michigan, USA

**Keywords:** Cladophialophora bantiana, 5-Aminolevulinic acid, Fluorescence, Phaeohyphomycosis, Case report

## Abstract

**BACKGROUND AND IMPORTANCE:**

Cerebral phaeohyphomycosis caused by *Cladophialophora bantiana* carries a high mortality rate of approximately 70%. Prompt, complete resection is associated with improved patient outcomes, with surgeons typically relying on imaging for appropriate margins of resection. 5-aminolevulinic acid (5-ALA) has traditionally been used in patients with gliomas; however, when used in our case of a patient with *C. bantiana* mimicking glioma, 5-ALA illuminated the capsule, allowing for optimal excision.

**CLINICAL PRESENTATION:**

An 81-year-old immunocompetent woman presented with dizziness, gait imbalance, headache, and left homonymous hemianopsia and was found to have right occipital hyperintensity concerning for malignancy. Subsequent craniotomy with 5-ALA fluorescence was performed, and the capsule was fully resected. On histopathological analysis, the sample revealed *C. bantiana*. Infectious disease was consulted, and the patient was treated with appropriate antifungals. Postoperatively, the fungus has completely resolved with no reported complications.

**CONCLUSION:**

*C. bantiana* is a fungal infection with high mortality and poor prognosis, capable of invading immunocompetent hosts. In our case, the intraoperative use of 5-ALA allowed for optimal surgical excision and no postoperative complications to date. This study suggests that 5-ALA could be used in situations not restricted to glioma to promote decreased mortality and improved patient outcomes.

**ABBREVIATIONS:**
5-ALA: 5-aminolevulinic acid
*C. bantiana*
: *Cladophialophora bantiana*.

Cerebral phaeohyphomycosis caused by *Cladophialophora bantiana* is a highly lethal fungal infection, despite current treatment interventions. Despite combined antifungal and surgical therapy, *C. bantiana* infections are associated with a mortality rate of approximately 70%.^1^ Unlike other well-known intracerebral fungi, *C. bantiana* is not limited to immunocompromised patients and presents significant challenges in neurosurgery because of its clinical and radiological resemblance to glioma. *C.* bantiana*-*induced lesions commonly mimic metastatic tissue, resulting in a typical diagnosis occurring intraoperatively or after autopsy.^2^ Given these clinical similarities, *C. bantiana* is frequently misdiagnosed in the early stages, leading to delays in initiating appropriate antifungal therapy until the infection has significantly progressed. In addition, secondary to the lack of published cases with consistent and successful therapy, there is yet to be standardized treatment of *C. bantiana* brain abscesses and antifungal treatments have only showed modest effects.^3^ Complete resection of the abscess capsule may result in improved patient outcomes as compared with aspiration or partial resection.^3^ Furthermore, recent evidence has suggested a clinical benefit to using neuronavigational techniques such as intraoperative ultrasound to resect these lesions effectively.^3^ In addition to intraoperative ultrasound, fluorescence imaging has clearly demonstrated utility in the operating room because of its capacity to enhance contrast and delineate tumor margins, thereby facilitating more precise surgical resection.^4^ Widely used to aid the resection of neoplastic tissue, 5-aminolevulinic acid (5-ALA) is often used to visualize suspected high-grade glioma and metastatic lesions.^5,6^ Therefore, 5-ALA may hold promise for visualizing additional diseases requiring excellent margins of resection, such as cerebral phaeohyphomycosis.

We present the case of an 81-year-old woman initially evaluated for a differential diagnosis of high-grade glioma vs metastatic lesion, who was ultimately diagnosed with a *C. bantiana* abscess. This report highlights the successful use of 5-ALA for clear intraoperative visualization and optimal resection of *C. bantiana–*induced lesions, resulting in the patient's full recovery after targeted antifungal therapy.

## CLINICAL PRESENTATION

**FIGURE 1.**
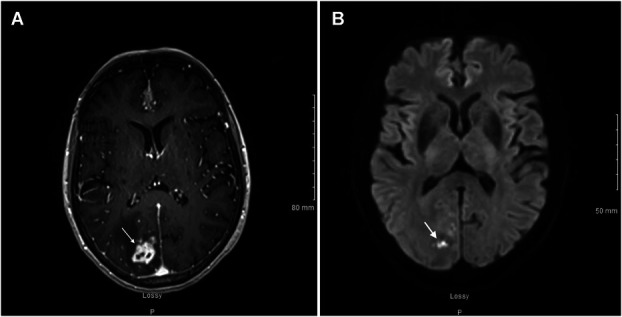
Axial T1 MRI postcontrast **A**, before abscess resection showing an irregular, enhancing intra-axial mass in the right parasagittal occipital lobe measuring 2.2 × 1.6 × 1.9 cm (white arrow), as well as an axial diffusion-weighted imaging **B,** showing a few regions of punctate diffusion restriction (white arrow).

An 81-year-old woman presented with a sudden onset headache and dizziness for several weeks. On examination, patient had left homonymous hemianopsia and gait imbalance. MRI with and without contrast was obtained and confirmed an irregular enhancing mass in the parasagittal right occipital lobe measuring 2.2 × 1.6 × 1.9 cm with surrounding vasogenic edema, concerning for primary neoplasm vs metastasis (Figure 1). The patient has a medical history notable for malignant breast cancer and squamous cell carcinoma of the skin; however, she remained immunocompetent and the chest, abdomen, and pelvis were not suggestive of extracranial primary site.

**FIGURE 2.**
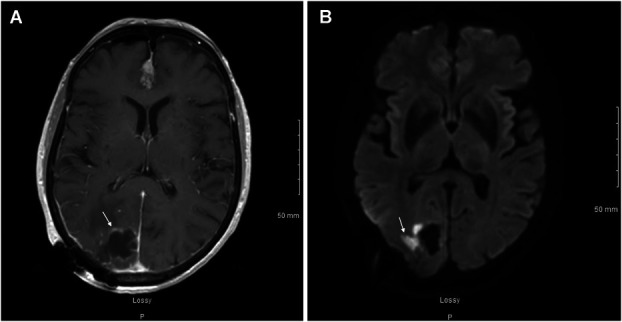
Axial T1 MRI postcontrast **A**, postresection showing the postoperative cystic cavity demonstrating some mild peripheral enhancement and a small, lateral, nodular area of enhancement (white arrow), as well as a postresection axial diffusion-weighted imaging **B,** demonstrating small areas of diffusion restriction on the lateral side of the cavity, largest measuring up to 1.5 cm (white arrow).

**FIGURE 3.**
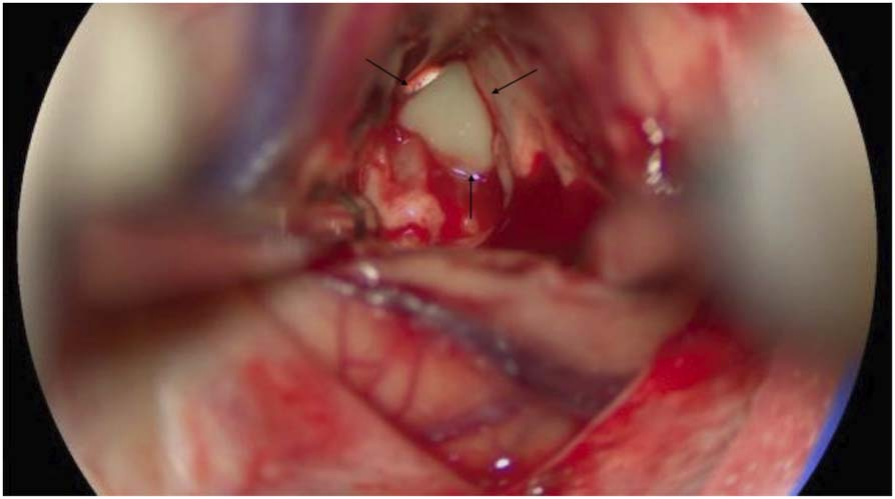
Intraoperative image demonstrating grossly thick, purulent drainage (black arrows), characteristic of liquid necrotic debris at the center of the indurated cranial mass consistent with an organized abscess capsule, clearly demarcated from the surrounding brain parenchyma.

**FIGURE 4.**
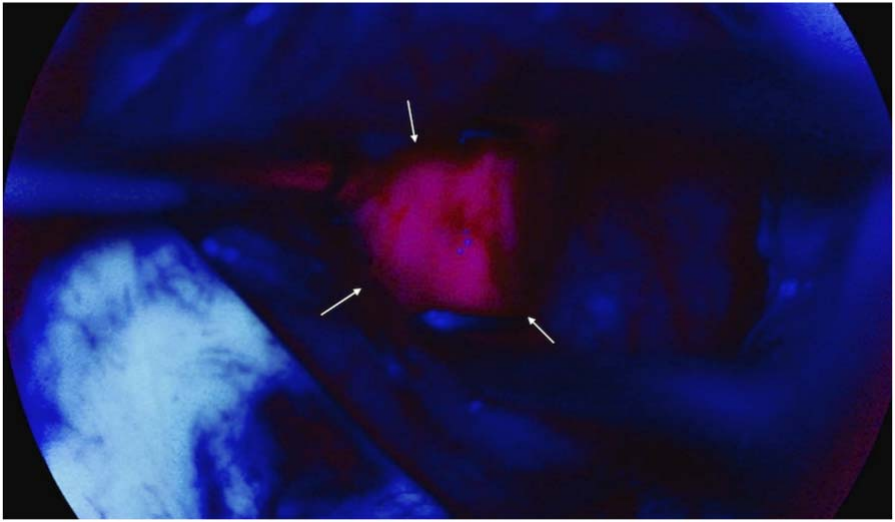
Intraoperative abscess capsule enhanced with 5-aminolevulinic acid fluorescence. After administration of 5-ALA, the abscess capsule demonstrated distinct pink fluorescence (white arrows). The surrounding blue fluorescence represents normal brain parenchyma of the occipital lobe, clearly delineating the inflammatory and healthy tissue.

The patient underwent a stealth-guided right occipital craniotomy and achieved complete lesional resection (Figure 2) with the aid of intraoperative 5-ALA fluorescence guidance. On resection, purulent drainage was immediately noted originating from the lesion's center (Figure 3). Most notably, the capsule exhibited a robust fluorescent signal with 5-ALA (Figure 4), which allowed for increased surgical precision and complete resection. Of note, ALA is indicated as an adjunct for the visualization of malignant tissue during surgery. This submission describes the use of this compound in the visualization of a fungal abscess capsule.

**FIGURE 5.**
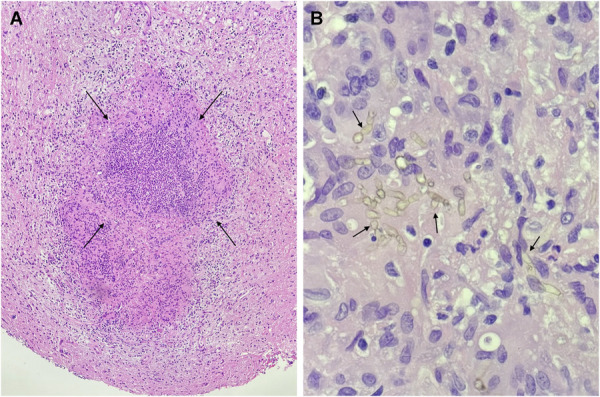
A hematoxylin and eosin–stained permanent section at 40× magnification **A,** demonstrating one of numerous abscess cavities (black arrows), as well as a 400× hematoxylin and eosin–stained permanent section **B,** demonstrating brown-pigmented, dematiaceous fungal hyphae (black arrows), proven to be *Cladophialophora bantiana*.

Histopathological analysis revealed numerous dematiaceous fungal hyphae, inflammatory cells, and fungal elements, findings most consistent with a fungal cerebral abscess (Figure 5). Multiple operative cultures subsequently yielded growth of mold identified as *C. bantiana*. The patient was tested negative for HIV and remained immunocompetent. On interview, she reported potential environmental exposure associated with a recent move to a new home in Florida, though no other risk factors of infection were identified.

Infectious disease was consulted, and intravenous liposomal amphotericin B therapy was initiated. This was followed by the addition of oral posaconazole 6 days later as per Infectious Disease recommendations, with a proposed 6-month course of antifungal therapy. The patient has progressed well postoperatively, with no neurological deficits aside from left homonymous hemianopsia and has not demonstrated recurrence to date. The appropriate informed consent was obtained, and approval from the Institutional Review Board was waived given the retrospective nature of this study.

## DISCUSSION

*C. bantiana* is a fungal infection that can affect immunocompetent individuals and carries a poor prognosis with a high mortality rate, regardless of a patient's immune status. Laboratory diagnosis typically requires over a week and involves culturing the pathogen as well as histopathological analysis. Studies indicate that delays in diagnosis notably contribute to patient mortality.^7^

Moreover, due to its rarity, no standardized antifungal treatment protocol currently exists; management thus far has primarily relied on limited case studies.^8^ Historically, treatment has involved combinations of agents such as amphotericin B, micafungin, voriconazole, flucytosine, and posaconazole, yielding variable outcomes; however, most have demonstrated synergistic or additive effects.^9^ This underscores the significance of a complete recovery after surgical intervention and antifungal therapy with the combination of liposomal amphotericin B and posaconazole.

In recent years, 5-ALA has been clinically shown to produce intracellular tumor-specific fluorescence through tumor-specific uptake of the protoporphyrin (PpIX) fluorescent molecule and is widely used to aid in the resection of neoplastic tissue.^4-6^ 5-ALA fluorescence has been reported with demyelinating disease, brain abscess and neurocysticercosis.^10^ Solis and Hansen first describe the use of 5-ALA in fungal abscesses. They detail a 54-year-old patient presenting with dysarthria and left facial droop with imaging suggestive of high-grade primary intracerebral neoplasm. In using 5-ALA, the abscess fluoresced pink whereas the surrounding healthy brain tissue remained blue, further suggesting a diagnosis of glioma. However, histopathological analysis confirmed Cryptococcus infection, and the patient was started on antifungal therapy. The 4-month follow up showed no remaining evidence of intracerebral infection, and there were no significant neurologic deficits at 9 months postresection.^11^ Aside from this report, the literature regarding intraoperative 5-ALA in the context of intracerebral fungal abscesses is extremely limited. To date, 5-ALA fluorescence of *C. bantiana* has not been documented. Meanwhile, in 2017, Aljuboori et al reported treatment of *C. bantiana* using antifungals and surgical resection without intraoperative 5-ALA fluorescence. They report a 59-year-old man with bilateral frontal ring enhancing lesions initially thought to be bacterial abscess but later confirmed as *C. bantiana*. The patient had undergone stereotactic aspiration with enlargement of abscesses 1 week later, followed by attempted aggressive total resection. Several months later, the patient showed enlarging lesions and underwent repeat surgical debridement and further antifungals, which ultimately resolved the fungal infection.^12^ This is consistent with several other incidences of worsening abscesses after surgical resection of *C. bantiana*, highlighting the importance of complete resection of the abscess capsule in preventing serial surgeries. As described in our case, 5-ALA successfully highlighted the fungal abscess capsule with impressive clarity. The use of 5-ALA in malignant glioma resection is widely recognized to facilitate significantly higher rates of complete resection, enhancing surgical precision and outcomes.^13^ Therefore, we propose that 5-ALA indication for non-neoplastic lesion resection be considered to support comprehensive removal and promote improved patient outcomes.

## CONCLUSION

In summary, we present a rare case of intracerebral *C. bantiana* abscess in an 81-year-old immunocompetent patient, mimicking high-grade glioma, who achieved full recovery after surgical resection with 5-ALA fluorescence and combination liposomal amphotericin B and posaconazole antifungal therapy. There have been no reported complications, and she has experienced a favorable postoperative course. This underscores the potential of 5-ALA fluorescence as a valuable adjunct in the surgical management of non-neoplastic lesions, particularly in future cases of *C. bantiana* among other intracerebral fungal abscesses. By expanding the use of 5-ALA beyond malignant gliomas, we may enhance surgical morbidity and mortality and patient outcomes in similarly complex cases, reinforcing its role as a standard tool in the resection of select non-neoplastic lesions.
